# Protective Factors of Nurses’ Mental Health and Professional Wellbeing During the COVID-19 Pandemic: A Multicenter Longitudinal Study

**DOI:** 10.3389/ijph.2024.1607449

**Published:** 2024-07-26

**Authors:** Jonathan Jubin, Line Martin, Naomi Kabwiku, Philippe Delmas, Ingrid Gilles, Annie Oulevey Bachmann, Claudia Huber, Marie-Chantal Loiselle, Jessica Rassy, Francisco Sampaio, Ricardo Salgado, Claudia Ortoleva Bucher

**Affiliations:** ^1^ La Source School of Nursing, HES-SO University of Applied Sciences and Arts Western Switzerland, Lausanne, Switzerland; ^2^ Lausanne University Hospital, Lausanne, Switzerland; ^3^ School of Health Sciences Fribourg, HES-SO University of Applied Sciences and Arts Western Switzerland, Fribourg, Switzerland; ^4^ School of Nursing, Faculty of Medicine and Health Sciences, University of Sherbrooke, Sherbrooke, QC, Canada; ^5^ Nursing School of Porto, Porto, Portugal; ^6^ CINTESIS@RISE, Nursing School of Porto, Porto, Portugal

**Keywords:** COVID-19 pandemic, nurses, mental health, professional wellbeing, protective factors

## Abstract

**Objective:**

Using a salutogenic approach, this study aimed to identify similarities in the protective factors of nurses’ psychological Quality of Life (QoL) and professional wellbeing (PWB) in four countries and to assess their variability over time during the COVID-19 pandemic.

**Methods:**

This multicentric study used a longitudinal design with three measurements points: Autumn 2021, spring 2022, and autumn 2022. The study consisted in a self-administered online questionnaire addressed to nurses working in hospitals. Across all measurement times, 3,310 observations were collected in France, 603 in Switzerland, 458 in Portugal, and 278 in Canada. The outcomes were psychological QoL and PWB, and several potential protective factors were used as determinants.

**Results:**

Analyses revealed few changes over time in the outcomes. Across all countries, psychological QoL was associated positively with resilience and perceived social support, whereas PWB was associated positively with the ability to provide quality work and support from colleagues and superiors.

**Conclusion:**

The findings of this study highlighted the potential of several factors protective of nurses’ psychological QoL and PWB. These should be fostered through policies and measures to support nurses.

## Introduction

In 2020, the World Health Organization declared a state of pandemic health emergency due to the spread of COVID-19. The virus caused 774 million cases worldwide and more than 7 million deaths as of March 2024 [1]. Strict measures had to be taken in many countries around the world, such as lockdown and social distancing, to contain the pandemic. This situation has led to a significant increase in hospital admissions and deaths, putting a strain on healthcare systems and healthcare professionals, nursing staff in particular.

Before the pandemic, nurses were already exposed to numerous challenges in their working environment [2, 3]. High proportions of administrative work, daily contact with others’ suffering, conflicts linked to professional roles and the need to hide emotions in their daily practice are characteristics of the nursing profession that place them at risk of various occupational health issues, such as high levels of stress [4–8]. During the pandemic, they were exposed to various additional stressors such as performing unusual tasks, equipment and medicines shortages, risk of contracting the virus, uncertainty linked to potential damage to one’s own or loved ones’ health, unusual patient deaths, reorganization of work spaces and schedules, and frequent changes in pandemic related policies [9–13]. These could have presented a risk of damage to their health [14]. Several studies highlighted individuals’ reactions to stress such as anxiety, insomnia, burnout and even post-traumatic stress [11, 12, 15–17]. These detrimental outcomes may persist over time, leading to persistent and disabling long-term consequences on quality of life (QoL), health, performance and job satisfaction, which could generate an important turnover [13, 18]. This turnover creates major problems for healthcare institutions, due to its negative impact on quality of care and patient safety, and their associated financial loss [18–22].

The majority of research took interest in factors susceptible to deteriorate nurses’ health. Yet, in a crisis situation, such as a pandemic, exposure to such factors is unavoidable. In this situation, it is equally important to investigate factors that might protect nurses’ health, as advocated by the salutogenic approach. The term salutogenesis was introduced by Aaron Antonovsky in 1979 as follows: a global orientation that expresses the extent to which one has a pervasive, enduring through dynamic feeling of confidence that one’s internal and external environments are predictable and that there is a high probability that things will work out as well as can reasonably be expected [23]. According to the salutogenic perspective, health can be assessed in a positive way, using QoL and wellbeing as indicators which, depending on a person’s ability to respond effectively to stressors, may remain stable even in the face of a crisis, as explained by Neuman’s System Model [11, 15, 24–27]. For example, nurses described in some studies the fact that they were able to discover internal resources of which they had not previously thought themselves capable, experiencing a sense of pride and professional growth that improved their QoL [14, 28]. This approach opens the possibility to develop recommendations that can be applied even when the context is a source of major stressors that cannot be avoided, such as a pandemic situation.

The present study focused on two nurses outcomes that are particularly at risk during a health crisis: psychological QoL and professional wellbeing (PWB) [29, 30]. It investigated the main protective factors from exposure to stressors identified in the literature, that could help protect their QoL during the pandemic [30–32]. These variables were i) resilience, ii) social support, iii) post traumatic growth, and iv) socio-professional factors. Resilience is defined as a process of positive adaptation in the face of an undesirable event, trauma, threat, or any source of significant stress [33]; Social support is defined as the support received by a person from their entourage to help them to manage and cope with stressors from their environment [34–36]; Post-traumatic growth is the individual’s ability to move beyond adaptive functioning, awareness, beliefs and goals shattered by a traumatic event [37]; and finally, the socio-professional factors in which we took interest as potential protective factors were support from nurses’ management hierarchies and colleagues, as well as their assessment of the quality of the work they were able to provide [38, 39]. The objective of the study was (i) to identify similarities in the protective factors of psychological QoL and PWB in several countries and (ii) to assess their variability over time.

## Methods

### Design and Population

Data collection took place in four different countries: France, Switzerland, Portugal, and Canada (Québec). A longitudinal design was used with the three measurements points: Autumn 2021 (A21), spring 2022 (S22), and autumn 2022 (A22). The data analyzed in the present article come from the INF+COVID-19 study that monitored nurses’ health, wellbeing and their protective factors from spring 2021 to autumn 2022 [40]. Though the protocol article only describes the Swiss part of the study [40], it was expanded to several other countries following the methodology as closely as possible. Convenience sampling was used, as we expected it would be difficult to obtain responses from nurses in a time of crisis such as the COVID-19 pandemic, and all nurses currently working in hospitals of the countries where the study took place were eligible to participate, regardless of the types of hospitals and services in which they worked. Yet, due to local legislations and access to nurses, recruitment methods were different in each country. Additionally, no data was collected in Portugal and Canada in spring 2021. For this reason, the present article focuses on three data collections: A21, S22, and A22 represented in Table 1. Its goal was not to compare samples and identify differences, as it would not be possible to explain them with such diverse contexts, but to try and identify recurrent associations that were consistent across these contexts.

**TABLE 1:** Data collections of the present study and number of respondents in each country (France, Switzerland, Portugal, and Canada, 2021-2022).

	2021-09	2021-10	2021-11	2021-12	2022-01	2022-02	2022-03	2022-04	2022-05	2022-06	2022-07	2022-08	2022-09	2022-10	2022-11
France	A21: 1264						S22: 1271						A22: 775		
Switzerland	A21: 294						S22: 183						A22: 126		
Portugal			A21: 336										A22: 122		
Canada		A21: 211							S22: 64					A22: 60	

For all countries, participants had access, through a link on the first page of the questionnaire, to an information sheet explaining the study, risks and benefits of participating, and providing consent information. The first screen of the questionnaire informed them that they needed to read the information sheet and proceed only if they accepted its terms. At the end of the first questionnaire, participants were asked if they agreed to be contacted for subsequent data collections and, if so, to provide an e-mail address. When these subsequent data collections happened, participants were contacted by e-mail and received three reminders at 1-week intervals as long as they had not participated. The data was stored at all times on a secure institutional server, and only the members of the project team in charge of the data management and analyses had access to the raw data.

#### France

In spring 2021, an invitation to participate in the study accompanied by a link to the online self-administered questionnaire was sent to all registered nurses through the French National Order of Nurses, followed by three weekly reminders. In total, 5,483 nurses working in hospitals filled the questionnaire. Among them, 3,457 accepted to be recontacted for the subsequent data collections. In A21, S22, and A22, these 3,457 participants were contacted by the research team with an e-mail inviting them to fill the questionnaire again followed by three weekly reminders. In A21 (September to November), 1,264 (36.6%) filled the questionnaire, 1,271 (36.8%) filled it in S22 (March to April), and 775 (22.4%) filled it in A22.

#### Switzerland

Nurses from 8 hospitals in western Switzerland (French-speaking) that accepted to relay the study were invited by their management to fill the questionnaire in spring 2021 and 625 participated. In A21 (September to November), 345 nurses from spring 2021 were recontacted and 153 (44.3%) participated. Moreover, 7 new hospitals’ management from central and eastern Switzerland (6 German-speaking and 1 bilingual) invited their nurses to take part in the study at this point. These 7 hospitals did not join the study in spring 2021, because of the time-sensitivity of the study. Indeed, collecting data as soon as possible during the pandemic was crucial to assess its impact and the data collection was quicker to start in French-speaking Switzerland. This resulted in a total 294 participants (141 from German-speaking and bilingual Switzerland and 153 from French-speaking Switzerland) filling the questionnaire in A21. In S22 (March to May), 420 nurses were recontacted again and 183 (146 and 37; response rate: 43.6%) filled the questionnaire, and in A22, 126 (89 and 37; response rate: 30.0%) of the 420 filled it again.

#### Portugal

The A21 (November 2021 to January 2022) recruitment was carried out via the website of the Portuguese Council of Nurses. In total, 336 nurses working in hospitals filled the questionnaire and 263 accepted to be recontacted at later data collections. No data collection was carried out in S22 in Portugal. In A22 (September to October), 122 participants (46.4%) filled the questionnaire.

#### Canada

In A21 (October to November), 5,000 nurses from Quebec were randomly selected from a list of nurses who accepted that their contact information can be transmitted to researchers and were invited to fill the online questionnaire in French. In total, 169 (3.4%) nurses working in hospitals filled the questionnaire and 140 accepted to be recontacted for later data collections. In S22 (May to June), 52 (37.0%) filled the questionnaire and in A22 (October to November), 49 (35.0%) filled it again.

### Measurements

#### Sociodemographic Variables

Several variables were measured: (i) gender; (ii) age; (iii) marital status; (iv) children; and (v) time spent in one’s current professional position. Additionally, degree of exposure to the COVID-19 was assessed with three modalities: “None” for no exposure to COVID-19 patients at the workplace; “Indirect” for participants who worked in services which received some COVID-19 patients but were not dedicated to it; and “direct” for participants who worked in services dedicated to treating COVID-19 patients.

#### Determinants

The perception of stress (proxy of the degree of exposure to stressors) was measured with the Perceived Stress Scale (PSS) [41] in its 10-items version, using a scale ranging from 1 (low perceived stress) to 5 (high perceived stress). It was translated into French, German, and Portuguese [41–43] This scale allows to measure the perception of everyday life situations as threatening. It has been used in the past with nursing staff in an epidemic context [44, 45].

Resilience was measured with the Connor-Davidson Resilience Scale (CD-RISC) [33] which includes 10 items rated from 1 (low resilience) to 5 (high resilience). This scale has been translated to French, German, and Portuguese [46–48].

The Multidimensional Scale of Perceived Social Support (MSPSS) [49] measures perceived social support through 12 items from three sources: family, friends and significant others. Each item is rated on a scale from 1 (low perceived support) to 7 (high perceived support). It was translated into French, German version, and Portuguese [34–36, 50].

The Post-Traumatic Growth Inventory–Short Form (PTGI–SF) [51, 52] measures positive psychological change following a traumatic event. It includes ten items rated from 1 (no psychological change) to 6 (many psychological changes). It was translated into French, German, Portuguese [51, 53, 54].

Three dimensions of the Copenhagen Psychosocial Questionnaire (COPSOQ) were used: Support from superior, support from colleagues, and quality of work [38, 39]. The first two factors are composed by three items each rated from 1 (low support) to 6 (high support) and the third is composed by two items rated from 1 (low quality) to 5 (high quality). This scale was translated into French, Portuguese, and German [38, 39, 55, 56]. Of note here, the Portuguese side of the study used the Job satisfaction dimension instead of the Quality of work dimension, which is close but not identical.

#### Outcome Variables

Psychological QoL was measured with the second dimension of the World Health Organization Quality of Life–BREF (WHOQOL-BREF) through 6 item*s* out of the 26 of the scale [57–59]. This scale was translated in French, German and Portuguese [57, 60]. Each item is rated from 1 (low QoL) to 5 (high QoL) by the participants. As the WHOQOL-BREF is widely used questionnaire, to ease comparisons between studies, mean scores were transformed as recommended by the authors to range from 0 (poor QoL) to 100 (good QoL) [58].

The Psychological Wellbeing Scale measures wellbeing with 8 items and was adapted by Fisher [61] to assess PWB. The scale measures self-perceived functioning in areas such self-esteem, purpose, relationships and it provides a reliable measurement of overall psychological wellbeing [62]. Items are rated from 1 (poor PWB) to 5 (strong PWB). This scale was already translated into French and German [63, 64], and was translated into Portuguese for the present study following Wild et al.’s methodology [65].

### Data Analysis

Descriptive statistics were computed with mean and standard deviation for numerical variables and frequencies for categorical variables. Cronbach’s α for all scales and at all measurement times were greater than 0.7. Then, missing values were replaced by the mean for numerical variables and by the mode for categorical variables to enable models comparisons. Cumulating all measurement points, there was 0.2% of missing data in France, 0.5% in Switzerland, 0.3% in Portugal, and 0.9% in Canada. For each country, after checking for normality, longitudinal random-intercept regression models with psychological QoL and PWB as outcomes were computed by adding variables block by block and assessing how each block improved the model. Categorical variables were processed as discrete variables. Block 1 included only the measurement point. Block 2 included sociodemographic variables. Block 3 included professional context variables. Block 4 included perceived stress and protective factors. Multicollinearity was assessed through the variance inflation factor, which was always smaller than 3.0. All analyses were performed with R 4.2.2 [66], using the packages lme4 v.1.1-31 [67] and lmerTest v.3.1-3 [68]. Significance level was set at *p* = 0.05.

## Results

### Descriptive Statistics

Descriptive statistics for categorical variables are presented in Table 2. In all countries and at all data collections, participants were mostly women (80.3%–91.8%). Participants were generally evenly distributed between all age categories and the proportion of older participants naturally increased at each data collection. Overall, approximately 70%–75% of participants lived in couple and 60%–65% had one or more children. Most participants occupied their current position for more than 5 years. This was especially salient in Portugal with more than 90% of participants occupying their position for more than 5 years. Approximately half of the participants were indirectly exposed to COVID-19 by working in non-specialized units that received COVID-19 patients and a quarter of them worked in direct contact with the disease in specialized units. This proportion was even higher in Switzerland where more than 40% of participants worked in direct contact with COVID-19.

**TABLE 2:** Descriptive statistics of respondents for categorical variables (France, Switzerland, Portugal, and Canada, 2021-2022).

	France			Switzerland			Portugal		Canada		
	A21	S22	A22	A21	S22	A22	A21	A22	A21	S22	A22
N	1,264	1,271	775	294	183	126	336	122	189	64	60
Categorical Variables (%)											
Gender: Woman	85.0	86.4	86.7	85.0	87.4	82.5	83.9	80.3	87.0	86.5	91.8
Gender: Man	14.7	13.5	12.9	13.9	12.0	17.5	15.8	18.0	11.8	13.5	8.2
Gender: Self-describe	0.2	0.1	0.3	0.0	0.0	0.0	0.0	0.8	0.6	0.0	0.0
Age: 18–29	16.7	10.9	9.5	21.8	10.4	8.7	12.2	7.4	16.6	11.5	14.3
Age: 30–39	26.3	24.0	21.5	28.6	33.9	28.6	32.7	32.0	29.0	34.6	22.4
Age: 40–49	29.7	31.6	29.9	27.2	31.7	31.7	32.4	28.7	24.9	26.9	32.7
Age: ≥50	26.8	32.7	38.2	22.1	24.0	31.0	22.6	31.1	29.6	25.0	28.6
Family situation: In Couple	75.1	75.0	72.8	70.4	71.0	74.6	69.6	70.5	71.0	71.2	69.4
Family situation: Single	21.8	20.7	23.1	26.9	23.5	20.6	23.2	20.5	26.0	23.1	26.5
Family Situation: Other	2.7	3.9	3.9	2.4	4.9	4.8	6.8	9.0	3.0	5.8	4.1
Children: Yes	64.8	67.6	68.4	51.4	65.0	62.7	64.0	66.4	59.8	61.5	59.2
Time in current position: <2 years	22.8	21.3	19.4	18.0	11.5	7.1	0.6	0.0	22.5	21.2	16.3
Time in current position: 2–5 years	30.7	28.3	24.5	25.9	25.1	27.0	8.9	2.5	26.0	21.2	26.5
Time in current position: >5 years	46.0	50.0	55.4	56.1	63.4	65.9	90.2	97.5	42.0	55.8	51.0
COVID-19 exposure: none	23.7	14.6	15.2	10.9	13.7	8.7	27.4	11.5	39.6	23.1	28.6
COVID-19 exposure: indirect	51.4	60.4	59.1	41.2	45.4	45.2	49.7	62.3	37.9	57.7	49.0
COVID-19 exposure: direct	24.5	24.5	24.7	47.6	41.0	46.0	22.6	26.2	22.5	19.2	22.4

Notes. Some percentages might not add up to 100 because of non-responses. A21: Autumn 2021; S22: Spring 2022; A22: Autumn 2022.

Descriptive statistics for numerical variables are presented in Table 3. As explained in the Methods section, comparing the differences between countries was not the object of the present study, as samples and data collections were too diverse. Thus, we will not analyse the descriptive statistics further.

**TABLE 3:** Descriptive statistics of respondents for numerical variables (France, Switzerland, Portugal, and Canada, 2021-2022).

	France			Switzerland			Portugal		Canada		
Numerical variables, mean (sd)	A21	S22	A22	A21	S22	A22	A21	A22	A21	S22	A22
Perceived stress (1–5)	2.99 (0.67)	3.02 (0.68)	2.98 (0.67)	2.93 (0.54)	2.81 (0.61)	2.71 (0.60)	3.03 (0.63)	2.90 (0.59)	3.07 (0.61)	2.77 (0.56)	2.77 (0.63)
Social support (1–7)	5.34 (1.26)	5.29 (1.30)	5.13 (1.41)	5.82 (1.02)	5.70 (1.07)	5.74 (1.11)	5.68 (1.08)	5.63 (1.15)	5.48 (1.19)	5.57 (1.30)	5.38 (1.03)
Resilience (1–5)	3.54 (0.67)	3.52 (0.70)	3.49 (0.70)	3.78 (0.55)	3.74 (0.59)	3.80 (0.63)	3.52 (0.69)	3.50 (0.69)	3.64 (0.64)	3.89 (0.54)	3.75 (0.59)
Post-traumatic growth (1–6)	3.25 (1.05)	3.16 (1.10)	3.14 (1.13)	3.30 (1.01)	3.44 (1.02)	3.43 (1.04)	2.90 (1.09)	2.93 (1.14)	3.36 (0.96)	3.43 (0.97)	3.09 (1.05)
Support from superiors (1–6)	3.66 (1.41)	3.68 (1.43)	3.64 (1.45)	4.25 (1.19)	4.26 (1.22)	4.37 (1.15)	3.10 (1.33)	3.06 (1.29)	3.81 (1.42)	4.13 (1.18)	4.18 (1.30)
Support from colleagues (1–6)	4.40 (1.02)	4.31 (1.12)	4.30 (1.09)	4.68 (0.78)	4.60 (0.88)	4.56 (0.78)	4.17 (1.00)	3.99 (0.94)	4.53 (0.94)	4.59 (0.98)	4.49 (0.95)
Quality of Work (1–5)	3.21 (0.90)	3.15 (0.95)	3.14 (0.95)	3.51 (0.84)	3.45 (0.88)	3.63 (0.95)	2.87 (0.92)	2.84 (1.01)	3.73 (0.89)	3.72 (0.93)	3.64 (1.00)
Psychological QoL (0–100)	57.8 (18.9)	56.1 (19.0)	56.7 (19.3)	66.8 (17.3)	67.2 (15.8)	68.4 (17.5)	63.7 (18.1)	65.0 (18.1)	61.3 (18.1)	65.0 (17.0)	63.7 (19.6)
Professional wellbeing (1–5)	3.68 (0.76)	3.59 (0.81)	3.57 (0.81)	4.05 (0.71)	4.01 (0.71)	4.05 (0.80)	3.72 (0.62)	3.71 (0.60)	3.82 (0.75)	4.01 (0.72)	3.90 (0.66)

Notes. QoL: Quality of life. A21: Autumn 2021; S22: Spring 2022; A22: Autumn 2022.

### Longitudinal Random-Intercept Regression Models

#### Psychological Quality of Life

Table 4 shows the results of the random-intercept regression models on psychological QoL and PWB for each country.

**TABLE 4:** Results of the longitudinal random-intercept regression models on psychological quality of life and professional wellbeing (France, Switzerland, Portugal, and Canada, 2021–2022).

	France		Switzerland		Portugal		Canada	
	Psy QoL	PWB	Psy QoL	PWB	Psy QoL	PWB	Psy QoL	PWB
Block 1	β	β	β	β	Β	β	β	β
Measurement point: S22	−0.03	−0.04*	0.02	0.01	-	-	−0.14	0.13
Measurement point: A22	0.01	−0.07**	0.01	−0.09	−0.01	−0.04	−0.10	0.03
Change in deviance	χ2 (2) = 6.64, *p* = 0.036	χ2 (2) = 18.20, *p* < 0.001	χ2 (2) = 3.50, *p* = 0.174	χ2 (2) = 0.33, *p* = 0.849	χ2 (1) = 1.73, *p* = 0.188	χ2 (1) = 0.02, *p* = 0.903	χ2 (2) = 0.36, *p* = 0.833	χ2 (2) = 5.53, *p* = 0.063
Block 2	β	β	β	β	β	β	β	β
Gender: Woman	−0.08*	−0.02	−0.04	−0.10	0.00	0.15	−0.26	−0.10
Gender: Self-describe	−0.32	−0.42	-	-	0.28	−0.02	−1.24	0.45
Age: 30–39	−0.01	−0.08	0.06	−0.05	−0.02	−0.09	−0.04	−0.07
Age: 40–49	−0.04	−0.15**	0.25*	−0.05	−0.03	−0.11	0.08	0.17
Age: ≥50	−0.05	−0.21***	0.28*	0.02	0.01	0.04	0.12	0.09
Family situation: Single	−0.05	−0.03	−0.19*	0.08	0.12	−0.12	−0.08	−0.08
Family Situation: Other	0.01	−0.08	−0.28*	−0.10	−0.06	0.37**	−0.08	0.02
Children: Yes	0.01	0.02	−0.06	−0.03	0.08	0.07	−0.01	0.04
Change in deviance	χ2 (8) = 24.10, *p* = 0.002	χ2 (8) = 18.27, *p* = 0.019	χ2 (7) = 19.46, *p* = 0.007	χ2 (7) = 3.52, *p* = 0.833	χ2 (8) = 19.35, *p* = 0.013	χ2 (8) = 18.04, *p* = 0.021	χ2 (8) = 22.20, *p* = 0.005	χ2 (8) = 15.20, *p* = 0.055
Block 3	β	β	β	β	β	β	β	β
Time in current position: 2–5 years	0.03	0.04	−0.13	0.12	0.19	−0.38	0.13	−0.01
Time in current position: >5 years	0.06	0.04	−0.14	0.12	0.24	−0.31	0.09	−0.07
COVID-19 exposure: none	0.00	0.07	−0.19*	−0.08	−0.03	−0.04	−0.11	0.09
COVID-19 exposure: indirect	−0.01	0.05	−0.10	−0.07	0.02	−0.06	−0.05	−0.04
Change in deviance	χ2 (4) = 2.20, *p* = 0.700	χ2 (4) = 8.118, *p* = 0.085	χ2 (4) = 9.74, *p* = 0.045	χ2 (4) = 1.79, *p* = 0.773	χ2 (4) = 0.53, *p* = 0.971	χ2 (4) = 2.39, *p* = 0.665	χ2 (4) = 3.15, *p* = 0.533	χ2 (4) = 9.59, *p* = 0.048
Block 4	β	β	β	β	β	β	β	β
Perceived stress	−0.42***	−0.26***	−0.31***	−0.13**	−0.48***	−0.24***	−0.43***	−0.37***
Social support	0.14***	0.01	0.22***	0.11**	0.25***	0.03	0.12*	0.09
Resilience	0.24***	0.13***	0.18***	0.09*	0.19***	0.21***	0.19***	0.03
Post-traumatic growth	0.08***	0.08***	0.08**	0.05	−0.03	0.02	0.08	0.07
Support from superiors	0.00	0.17***	−0.02	0.10**	−0.06	0.05	0.00	0.11*
Support from colleagues	0.05***	0.12***	0.07*	0.13***	0.04	0.17***	0.09*	0.05
Quality of Work	0.07***	0.28***	0.13***	0.35***	0.13***	0.34***	0.05	0.30***
Change in deviance	χ2 (7) = 2,229.60, *p* < 0.001	χ2 (7) = 1973.57, *p* < 0.001	χ2 (7) = 341.19, *p* < 0.001	χ2 (7) = 262.41, *p* < 0.001	χ2 (7) = 452.13, *p* < 0.001	χ2 (7) = 370.94, *p* < 0.001	χ2 (7) = 176.96, *p* < 0.001	χ2 (7) = 163.53, *p* < 0.001

Notes. *p* significance codes: 0 “***” 0.001 “**” 0.01 “*” 0.05. Psy QoL: psychological quality of life; PWB: Professional wellbeing; β: standardized regression coefficient from the complete model including all blocks. Reference levels: Measurement point vs. A21; Gender vs. Man; Age: vs. 18–29; Family situation vs. Couple; Children vs. No; Time in current position vs. <2 years; COVID-19, exposure vs. Direct.

Block 1 significantly improved the model quality for psychological QoL only in France data [χ_2_ (2) = 6.64, *p* = 0.036]. Psychological QoL was lower in A22 than it was in A21 (β = −0.06, *p* = 0.011). This effect did not hold in the complete model.

Block 2 improved the model significantly for all countries (χ_2_ (8) range: 19.35–24.10, p range: 0.013–0.002). In France, Portugal and Canada, women reported lower psychological QoL than men (β range: −0.19 to −0.62, p range: 0.005–0.002). Moreover, in Switzerland, psychological QoL tended to increase with age, the difference between 18–29 and 50+ reaching significance (β = 0.33, *p* = 0.040), and, in Switzerland and Canada to be worse in single participants than in those living in couple (β range: −0.32 to −0.46, p range: 0.006–0.001).

Block 3 only improved the model significantly for Switzerland data [χ_2_ (4) = 6.64, *p* = 0.036]. Participants who occupied their position for longer reported lower psychological QoL, the difference reaching significance between those who occupied their position for less than 2 years and those who did for 2–5 years (β = −0.24, *p* = 0.027), and barely failing to reach significance for those who occupied their post for more than 5 years (β = −0.23, *p* = 0.059).

Block 4 significantly improved the model for all countries [χ_2_ (7) range: 2,229.60–176.96, ps < 0.001]. Perceived stress was consistently and negatively associated with psychological QoL (β range: −0.31 to ‒0.48, ps < 0.001) whereas social support (β range: 0.12–0.25, p range: 0.015-< 0.001) and resilience (β range: 0.18–0.24, ps: <0.001) were positively associated with it. Psychological QoL was also associated positively with post-traumatic growth in France and Switzerland (βs = 0.08, p range: 0.007-< 0.001), with support from colleagues in France, Switzerland, and Canada (β range: 0.05–0.09, p range: 0.030-< 0.001), and with quality of work in France, Switzerland, and Portugal (β range: 0.07–0.13, ps < 0.001).

#### Professional WellBeing

As for psychological QoL, Block 1 significantly improved the model quality for PWB only with France data [χ_2_ (2) = 18.20, *p* < 0.020]. PWB was lower in S22 (β = −0.08, *p* = 0.001) and A22 (β = −0.12, *p* < 0.001) than it was in A21.

Block 2 improved the model significantly for France and Portugal (χ_2_ (8) range: 18.04–18.27, p range: 0.030–0.019). In France, women reported lower PWB than men (β = −0.14, *p* = 0.023). Additionally, in France 30–39 years old reported lower PWB than 18–29 (β = −0.15, *p* = 0.033), participants in a family situation other than single or in couple reported lower PWB than those in couple (β = −0.25, *p* = 0.008).

Block 3 only improved the model significantly for Canada data (χ_2_ (4) = 9.59, *p* = 0.048). Participants who were not exposed to COVID-19 in the line of their work reported higher PWB than those who were directly exposed by working in units dedicated to treating COVID-19 patients (β = 0.34, *p* = 0.030).

Block 4 significantly improved the model for all countries [χ_2_ (7) range: 1973.57–163.53, ps < 0.001]. Perceived stress was negatively associated with PWB across all countries (β range: −0.13 to ‒0.37, p range: 0.001-< 0.001), whereas quality of work (β range: 0.28–0.35, ps < 0.001) was consistently associated positively with PWB. This outcome was also associated with resilience (β range: 0.09–0.21, p range: 0.029-< 0.001) and support from colleagues (β range: 0.13–0.17, ps < 0.001) in France, Switzerland and Portugal; with support from superiors (β range: 0.10–0.17, p range: 0.028-< 0.001) in France, Switzerland and Canada; with social support in Switzerland (β = 0.11, *p* = 0.004); and with post-traumatic growth in France (β = 0.08, *p* < 0.001).

## Discussion

### Results Overview

The present longitudinal study used a salutogenic approach to measure the psychological QoL and PWB as well as the protective factors mobilized by nurses during the COVID-19 pandemic in Switzerland, France, Canada, Portugal. Nurses with a high level of perceived stress reported lower levels of psychological QoL and PWB. These results are in line with the literature on this period of crisis [15, 16, 21, 22, 28, 69, 70] and congruent with the findings of other studies carried out during epidemics [71]. During the COVID-19 pandemic, some authors have observed similar results, in terms of the moral and psychological impact of the various sources of stress caused by the pandemic situation [9, 11, 70]. As numerous studies showed that nurses were already exposed to stressors before the pandemic, which can put them in a situation of moral distress with a low level of psychological health [69].

Results highlighted that nurses’ resilience was positively associated with their psychological QoL and PWB during the pandemic in every country involved in the study, except for Canada. These findings are in line with other articles, which have studied these variables [4, 15, 69]. Resilience has also been shown to be a moderator of the association between stress and nurses’ mental health [72]. The current findings suggest a direct connection rather than moderation, possibly attributable to differences in the operationalization of perceived stress in this study. In another study, resilience was explained as a significant predictor of 16% of the variance in moral distress and 8% of somatic symptoms in nurses [11]. A wider study conducted in 17 countries showed that, generally speaking, people with the highest levels of resilience were those who considered their own mental health to be good or excellent, even though some aspects of their lives were just as disrupted by the pandemic as others [16]. Resilience would therefore act as a moderator of nurses’ potential psychological distress, making the link between a high level of resilience development and a reduction in symptoms suggestive of burnout, anxiety, depression, and psychological distress leading to sick leave or quitting the job or profession [11, 70, 71].

Based on the premise that resilience contributes to the development of post-traumatic growth through the implementation of positive coping strategies, it is necessary to consider interventions aimed at developing nurses’ resilience [14]. In the workplace, this process involves affective, cognitive and behavioral self-regulation processes, mobilizing internal (e.g., cognitive appraisal capacity or positive affect regulation) and external (job support or clinical supervision) resources that enable individuals to adapt and restore optimal functioning after stressful workplace events [73]. It might also be useful, at the individual level, to promote personalized self-care interventions and the development and maintenance of resilience to preserve nurses’ QoL the outset of their practice [11, 74–78].

Prior research has shown that social support also plays an important role as protective factors for nurses from negative outcomes caused by the pandemic and that it can help them to preserve their psychological QoL and their PWB [79, 80]. Our results confirmed these findings. Literature shows that social support (from family, friends, and significant others, as well as from colleagues and superiors) can help nurses, who might become second victims in case of critical events, to overcome potentially stressful events, alleviate symptoms of depression and anxiety, and maintain the health and wellbeing of nurses during a pandemic [18, 81]. The same study also found a significant association between social support and physical and psychological health [18]. Indeed, nurses benefiting from a high level of social support showed a significant reduction in burnout, better self-efficacy during the pandemic, as well as maintaining wellbeing [80, 82], improving job satisfaction [18] and motivation to stay in the profession [79].

Regarding nurses’ PWB during COVID-19 pandemic, our study identified perceived support from colleagues and superiors as a factor strongly associated with better PWB. This positive association is consistent with previous studies that found support from colleagues to be an important health resource for nurses at work [83]. It is interesting to note that some studies observed an association between nurses’ health, resilience, and perceived social support during the pandemic [30]. Establishing formal and supporting informal social support could thus be a way for institutions to foster nurses’ health by providing them with emotional and practical support. This can be achieved through several ways, such as peer support groups, mentorship programs, and opportunities for social interaction among staff (e.g., [84–86]). Additionally, supervisors might benefit from leadership training or interventions to provide better support to nurses [87].

In France, Switzerland, and Canada, the ability to provide quality work was also consistently and positively associated with PWB and, to a lesser extent, with psychological QoL. In Portugal, job satisfaction, in place of quality of work, showed the same associations. This finding is congruent with the literature, as research in palliative care nurses suggested that nurses delivering quality care were more satisfied and felt more accomplished [88, 89]. Moreover, a qualitative study showed that quality patient care was a central theme associated with nurses’ commitment to their work [90]. Nurses and institutions should thus establish dialogue to assess nurses’ satisfaction not only with their working conditions, but also with the quality of the care they provide as well as the resources at their disposal in terms of staffing, equipment, and time (e.g., [91]). These should be assessed regularly in order to promptly address any gaps.

### Limitations

The main limitation of this present study was the impossibility to directly compare the four countries in which measurements took place which was mainly due to the differences in recruitment methods which could not be harmonized. Additionally, the pandemic and the workload it generated might not have impacted nurses of all countries at the same time, leading to data collections happening in different contexts. Nevertheless, the fact that several associations were consistently observed despite these differences is of high interest, as this means that they are very robust. The second limitation was the high attrition rate between measurement times. This might be explained by the tough working conditions and work overload that nurses faced during this period, preventing them from having time to take part in such surveys. It is also possible that nurses showed less interest in the research project due to pandemic fatigue. They may have felt the need to step back and mentally detach from this traumatic event. Therefore, even though the research project could offer interesting perspectives, nurses may have chosen to focus on other aspects of their professional or personal lives to find balance and emotional renewal. Another limitation is that nurses who were in the worst health status might have left their job or been in sick leave, preventing them from participating in the survey. In consequence, the results might reflect only the situation of nurses in better health, as described by the healthy worker effect [92, 93]. Our results should thus be interpreted with caution, as they may present an overestimated level of nurses’ psychological QoL and PWB. Finally, as the questionnaire was self-assessed, biases such as social desirability might have arisen [94].

### Conclusion

Nurses are the pillar of health systems and were at the front line during the COVID-19 pandemic. The strain to which they have been subjected combined with the usual elevated stress context of the profession aggravated the already preoccupying staff shortages and turnover rate, making it urgent to assess which protective factors are better suited for large scale interventions. The present study highlighted several protective factors that consistently protected nurses’ psychological QoL and PWB across four countries throughout the COVID-19 pandemic. Thus, these factors appear to be relevant targets for interventions aiming to support nurses in crisis time. In order to better prepare potential epidemic or pandemic situations in the future, policymakers and care institutions should focus on how best to implement such interventions in their specific contexts, as healthcare systems are only as resilient as their nurses.
